# Phosphorylation of Parkin at serine 131 by p38 MAPK promotes mitochondrial dysfunction and neuronal death in mutant A53T α-synuclein model of Parkinson’s disease

**DOI:** 10.1038/s41419-018-0722-7

**Published:** 2018-06-13

**Authors:** Jialong Chen, Yixian Ren, Chen Gui, Menglan Zhao, Xian Wu, Kanmin Mao, Wenjun Li, Fei Zou

**Affiliations:** Department of Occupational Health and Occupational Medicine, School of Public Health, Southern Medical University, Guangdong Province, Guangzhou, 510515 China

## Abstract

α-synuclein abnormal accumulation and mitochondria dysfunction are involved in the pathogenesis of Parkinson’s disease. Selective autophagy of mitochondria (mitophagy) is a crucial component of the network controlling the mitochondrial homeostasis. However, the underlying mechanism that mutant α-synuclein induces mitochondrial abnormality through mitophagy impairment is not fully understood. Here, we showed that mutant A53T α-synuclein accumulation impaired mitochondrial function and Parkin-mediated mitophgy in α-synucleinA53T model. α-synucleinA53T overexpression caused p38 MAPK activation, then p38 MAPK directly phosphorylated Parkin at serine 131 to disrupt the Parkin’s protective function. The p38 MAPK inhibition significantly reduced cellular apoptosis, restored mitochondrial membrane potential as well as increased synaptic density both in SN4741 cells and primary midbrain neurons. These findings show that the p38 MAPK-Parkin signaling pathway regulates mitochondrial homeostasis and neuronal degeneration, which may be a potential therapeutic strategy of PD via enhancing mitochondrial turn-over and maintenance.

## Introduction

Parkinson’s disease (PD) is the most common neurodegenerative movement disorder characterized by bradykinsesia, muscular rigidity, rest tremor, and postural and gait impairment. Published evidence supports that α-synuclein aggregation and mitochondrial dysfunction are the main causes contributes to the progressive neuronal degeneration of PD^1^. Mutations in α-synuclein, including A53T mutant, result in autosomal dominant form of familial PD. The toxicity of α-synuclein accumulation affects cell organelles function including synaptic vesicles, mitochondria, ER, lysosomes, and autophagosomes^2^. However, it remains unclear whether and how α-synuclein aggregation involved in mitochondrial homeostasis.

Parkin, an ubiquitin E3 ligase, plays a neuroprotective role in clearing dysfunctional mitochondria through mitophagy, protects neurons from α-synuclein toxicity and kinase-induced excitotoxicity^3^. Loss-of-function of Parkin results in mitochondrial dysfunction, which is a core pathogenic process in PD. A variety of post-translational modifications of Parkin have been proved that affect its ubiquitin E3 ligase activation, including phosphorylation, ubiquitylation, and S-nitrosylation.^4,5^. Parkin’s function may be regulated by multiple factors. This warrants further study to better understand the underlying molecular mechanisms of mitophagy, the relationship between mitochondrial clearance machinery and PD pathogenesis.

As a member of MAPKs, the p38-mitogen-activated protein kinase (MAPK) signaling affects a variety of intracellular responses, with well-recognized roles in inflammation, cell-cycle regulation, cell death, development, differentiation, senescence, and tumorigenesis. The majority of studies investigating the role of p38 MAPK in normal ageing as well as neurodegenerative diseases focus on its function in the process of controlling neuroinflammation. p38 MAPK activation induces apoptosis through mitochondrial death pathway, contributes to neuronal degeneration^2^. In addition, p38 pathway-inhibited autophagy and impaired mitochondrial function in Parkin deficiency mice^3^. The deficiency of p38 MAPK significantly increased autophagy, attenuated amyloid pathology in AD models^6^. Therefore, we proposed a hypothesis that p38 MAPK may be a vital factor involved in mitophagy in PD.

We investigate that mitophagy impairment, caused by α-synuclein aggregation, is rescued by p38 MAPK inhibition. Here we show that Parkin is a substrate of p38 MAPK and p38 MAPK negatively regulates Parkin’s protective function. Phosphorylation of Parkin at serine 131 occurs under mutant A53T α-synuclein overexpression, and pharmacological inhibitor of p38 MAPK reduces the phosphorylation of Parkin serine 131, rescues the Parkin-mediated mitophagy. Our studies identify a novel mechanism which p38 MAPK contributes to α-synuclein-induced neuronal death by inhibiting the selective degradation of damaged mitochondria.

## Results

### p38 MAPK is activated in SNpc of α-synucleinA53T transgenic mice model

**Fig. 1:**
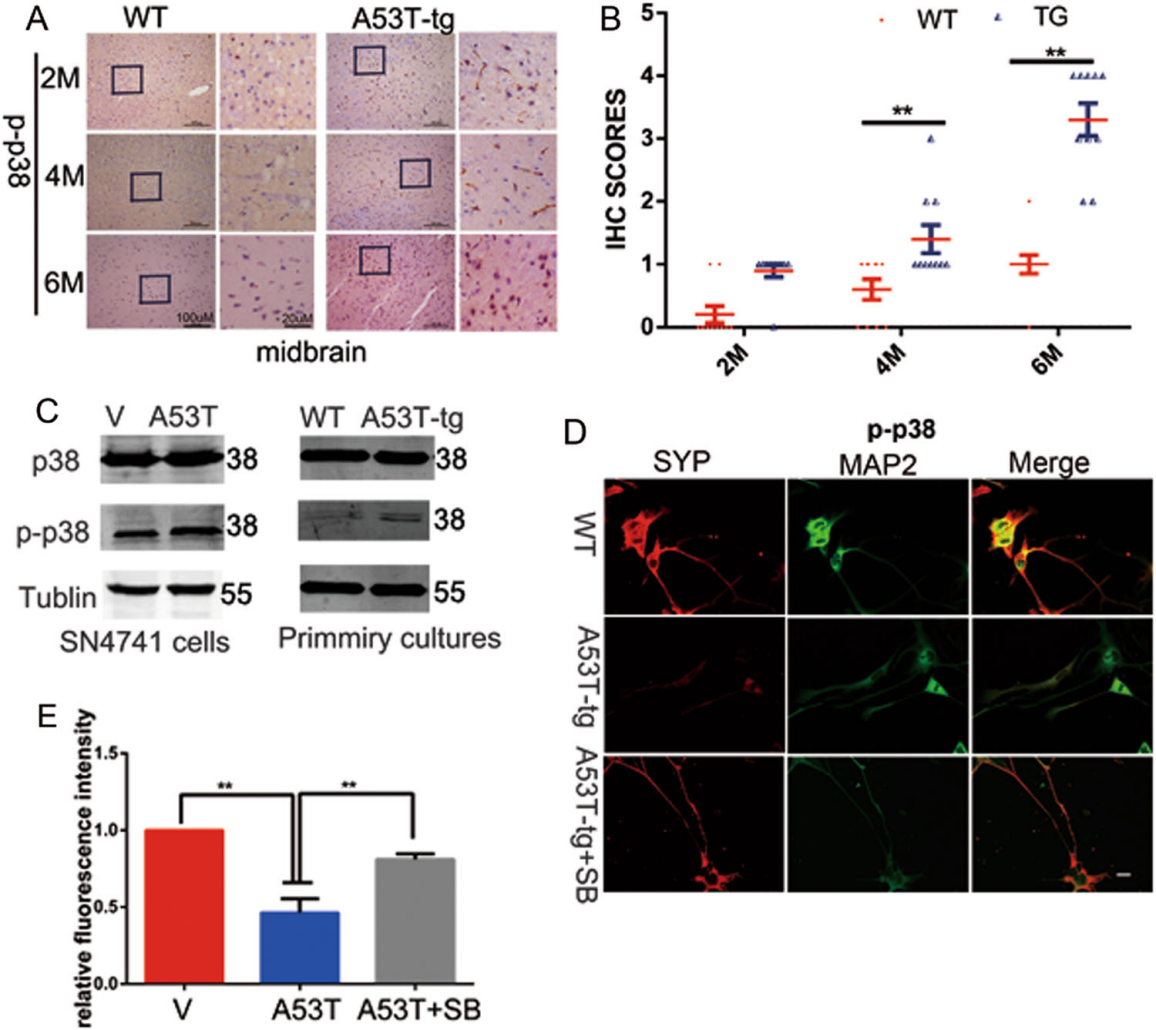
p38 MAPK is activated in SNpc DA neurons of α-synucleinA53T transgenic mice model. **a** Immunohistochemistry (IHC) staining of phosphorylated p38 in the midbrain of 2, 4, and 6 month α-synuclein^A53T^-tg or wild-type mice. **b** Statistical analysis of the average score of phosphorylated p38 staining between α-synuclein^A53T^-tg and wild-type mice. *p < 0.05 (Student’s *t*-test). **c** Cell lysates from primary midbrain cultures and SN4741 cells s were immunoblotted using the indicated antibodies to determine the levels of phosphorylated p38. Mean ± SEM. *n* = 3. *p < 0.05. (**d**, **e**) Primary midbrain cultures from wild-type mice and α-synuclein^A53T^-tg mice. Cultures of both genotype were grown in vitro, fixed, and immunostained for dendritic marker MAP2 and presynaptic marker SYP(synaptophysin) and shown in **e** as mean ± SEM. *n* = 3. *p *<* 0.05. The provided Scale bar in merge image represent 5 μm. Mean ± SEM. *n* = 3. *p < 0.05

α-synuclein, a major component of lewy body, is implicated in the pathogenesis of PD and related neurodegeneration. Mutation in α-synuclein (A53T) is responsible for autosomal dominant early-onset PD^7,8^. The role of p38 MAPK in neurodegenerative disorder is crucial because it triggers unwanted phenotypes such as microglia activation, neuroinflammation, oxidative stress, and apoptosis^9^. To address whether p38 MAPK activation contributes to PD pathogenesis, we used α-synucleinA53T transgenic mice (α-synuclein^A53T^-tg mice). First, we investigated the levels of p38 MAPK phosphorylation in the **SNpc** of 2, 4, and 6 month α-synuclein ^A53T^-tg mice by IHC. Results showed that compared with wild-type mice, p38 MAPK was activated in SNpc of SNCA^A53T^-tg mice (Fig. 1a, b). Next, we derived primary midbrain cultures from SNCA^A53T^-tg mice. The activation of p38 MAPK was further confirmed in SNpc neurons and α-synuclein A53T overexpression SN4741 cells in vitro (Fig. 1c). Additionally, the JNK and ERK phosphorylation showed no difference in α-synuclein A53T overexpression SN4741 cells (figure S1 D). To further identify whether p38 MAPK activation participated in the pathogenesis of PD, primary SNpc neurons were preincubated with p38 MAPK inhibitor SB203580 (24 h, 10 μM) at 8 DIV(days in vitro) and then quantified the synaptic density based on SYP (synaptophysin) at 10 DIV^10^. Interestingly, compared with the primary SNpc neurons from wild-type mice, the level of synaptic density of primary neurons from α-synuclein^A53T^-tg mice was significantly decreased, while SB203580 rescued this effect (Fig. 1d, e). These data suggested that p38 MAPK activation participates in the process of neuronal and synaptic loss of PD.

### Mitophagy is impaired in α-synucleinA53T SN4741 cells

**Fig. 2:**
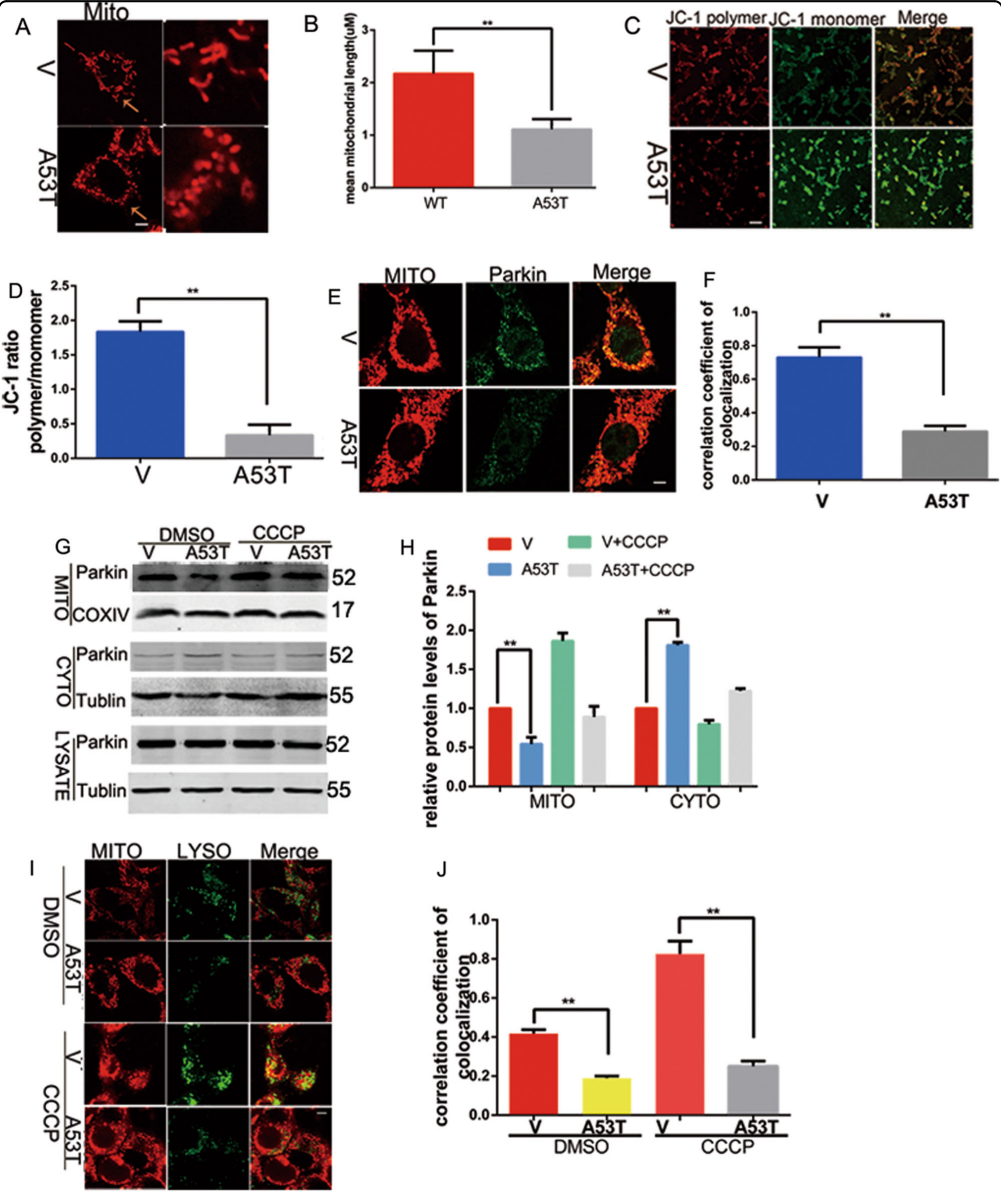
Mitophagy is impaired in α-synucleinA53T SN4741 cells. (**a**, **b**) Mitochondrial morphology was assessed using mitochondria tracker and shown in **b** as mean ± SEM. *n* = 10. *p *<* 0.05. The provided Scale bar in merge image represent 5 μm. (**c**, **d**) Mitochondrial membrane potential was assessed by JC-1 and shown in D as mean ± SEM. *n* = 3. *p *<* 0.05. The provided Scale bar in merge image represent 50 μm. (**e**, **f**) The co-localization analysis of mitochondria and Parkin with mitochondria tracker and Parkin antibody was examined under confocal microscope and shown in (**f**) as mean ± SEM. *n* = 10. *p *<* 0.05. (**e**, **g**, **h**) After treatment, total cell lysates, mitochondria-enriched, and cytosolic fractions were prepared. Immunoblotting analyzed the level of Parkin in the mitochondria component, cytosolic fractions, and total cell lysates. Tublin was used as loading control in total cell lysates, cytosolic fractions. COXIV was used as loading control in mitochondria. The data were performed in three times and shown in (**h**) as mean ± SEM. *n* = 3. *p *<* 0.05. (**i**, **j**) The co-localization analysis of mitochondria and lysosome with mitochondria tracker and lysosome tracker was examined by confocal microscope and shown in **j** as mean ± SEM. *n* = 10. *p *<* 0.05. The provided scale bar in merge image represent 5 μm

Overexpression of A53T mutation α-synuclein in nerve cell, resulting in α-synuclein accumulation, has been identified to induce PD-like neuropathology. SN4741 cells were transfected with A53T mutant α-synuclein. Fig. 2a–d showed that α-synucleinA53T led to mitochondrial fragmentation (Fig. 2a, b) and declined mitochondrial membrane potential (Fig. 2c, d). To verify whether α-synuclein accumulation affects the clearance of damaged mitochondria, the level of Parkin in mitochondria and cytosol were measured. SN4741 cells were transfected α-synucleinA53T, treated with DMSO or CCCP(10 μm, 6 h). We performed a confocal microscopy analysis using antibodies against Parkin and Mito Tracker Red. Indeed, the Parkin translocation to mitochondria was significantly decreased after α-synucleinA53T overexpression (Fig. 2e, f). To further confirm these results, immunoblotting showed that α-synuclein A53T overexpression decreased the portion of Parkin in mitochondrial components, while increased in cytosol (Fig. 2g, h). Meanwhile, α-synucleinA53T increased mitochondrial PINK1. (figure S1D). In order to examine the change of mitochondria and lysosomes fuse to mitochondrial autolysosomes, we stained the mitochondria and lysosomes with Mito Tracker Red and Lyso Tracker Green, respectively. Trackers staining showed that α-synuclein accumulation decreased the formation of mitochondrial autolysosomes (Fig. 2i, j), which indicated the occurrence of mitophagy was inhibited. These results suggested that α-synuclein aggregation causes mitochondrial dysfunction as well as mitophagy impairment.

### Stimulation of mitophagy by p38 MAPK inhibitor SB203580 alleviates mitochondrial dysfunction and cell death

**Fig. 3:**
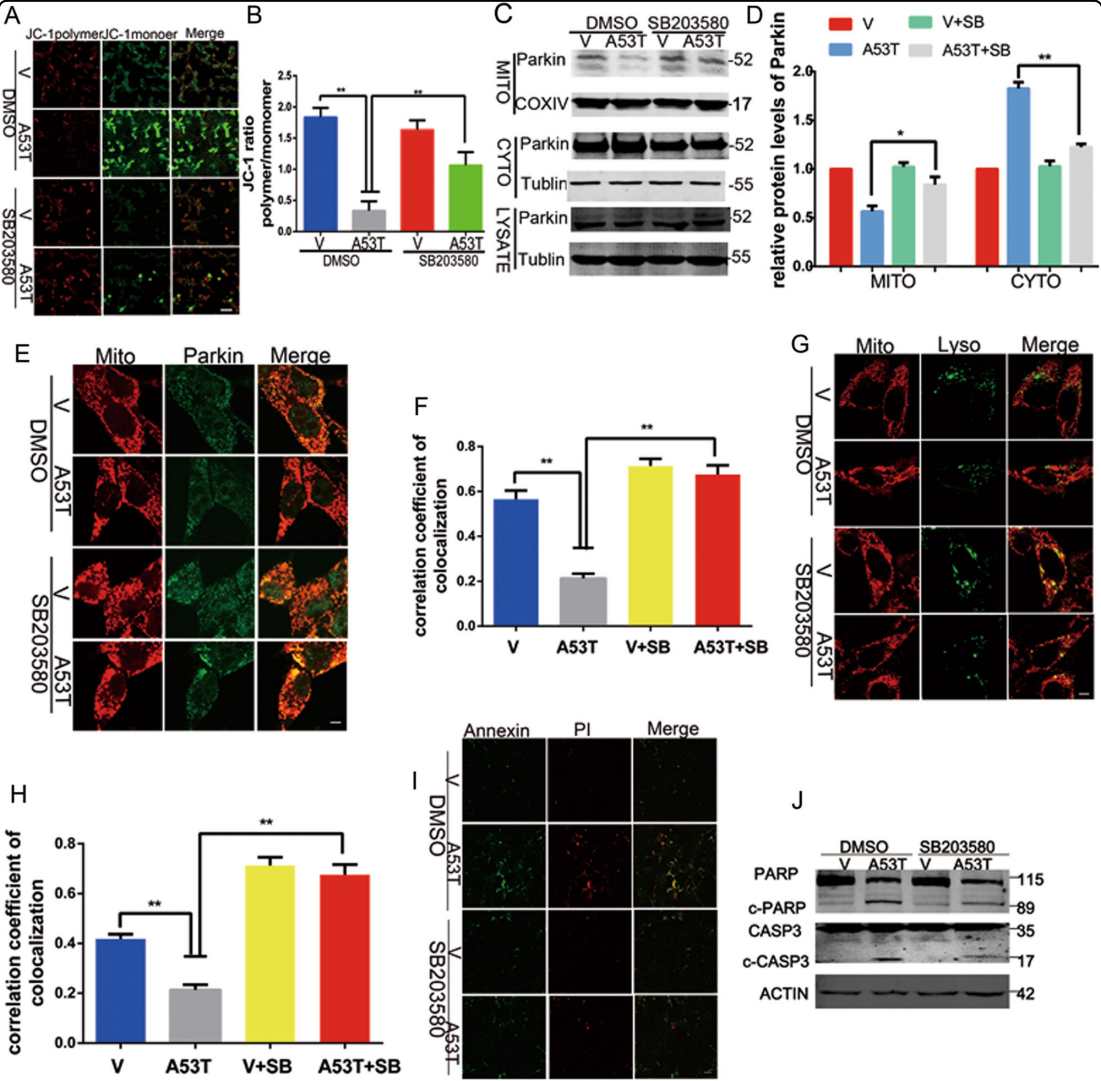
Stimulation of mitophagy by p38 MAPK inhibitor SB203580 alleviates mitochondrial dysfunction and cell death. (**a**, **b**) Mitochondrial membrane potential was assessed by JC-1 and shown in B as mean ± SEM. *n* = 10. *p *<* 0.05. The provided Scale bar in merge image represent 50 μm. (**c**, **d**) Cells transfected with empty vector, **α**-synuclein A53T were treated with DMSO or SB203580 (10 μM, 24 h). The level of Parkin in the mitochondria component, cytosolic fractions, and total cell lysates were measured by the indicated antibodies. Tublin was used as loading control in total cell lysates and cytosolic fractions, COXIV was used as loading control in mitochondria and shown in (**d**) as mean ± SEM. *n* = 3. *p < 0.05. (**e**, **f**) The co-localization analysis of mitochondria and Parkin with mitochondria tracker and anti-Parkin was detected by confocal microscope and presented in (**f**). Mean ± SEM. *n* = 10. *p < 0.05. The provided Scale bar in merge image represent 5 μm. (**g**, **h**) The co-localization analysis mitochondria and lysosome with mitochondria tracker and lysosome tracker was examined by confocal microscope and presented in (**h**). Mean ± SEM. *n* = 10. *p < 0.05. The provided Scale bar in merge image represent 5 μm. **i** Representative images of Annexin (green) and PI (red) double fluorescence staining showing cell apoptosis. The provided Scale bar in merge image represent 50 μm. **j** Immunoblotting analyzed the level of apoptotic caspases 3 or poly (ADP-ribose) polymerase and mean ± SEM. *n* = 3. *p < 0.05

In order to examine whether p38 MAPK involved in mitochondrial dysfunction in PD, we treated α-synucleinA53T overexpression SN4741 cells with p38 MAPK inhibitor SB203580 (10 μm, 24 h). First, we investigated mitochondrial membrane potential by JC-1 staining. The results showed that p38 MAPK inhibitor SB203580 significantly reduced mitochondrial membrane potential loss caused by α-synucleinA53T (Fig. 3a, b). Furthermore, we measured the level of mitophagy. Both western blot and immunofluorescence revealed that SB203580 increased Parkin translocation to mitochondria (Fig. 3c–f). SB203580 also enhanced the co-localization of mitochondria and lysosome (Fig. 3g, h), suggesting that the increasing of the formation of mitochondrial autolysosomes. Besides, SB203580 markedly decreased the cell apoptosis rates through detecting the levels of cleavage of apoptotic caspases 3 and poly (ADP-ribose) polymerase, as well as FITC-Annexin V and PI staining (Fig. 3i, j). Meanwhile, SB203580 did not affect α-synuclein mRNA expression (fig. S1 G, H). Together, these data suggested that p38 MAPK inhibitor plays a protective role from α-synuclein aggregation via stimulation of mitophagy.

### SB203580 treatment inhibits mitochondrial dysfunction through regulating Parkin activation

**Fig. 4:**
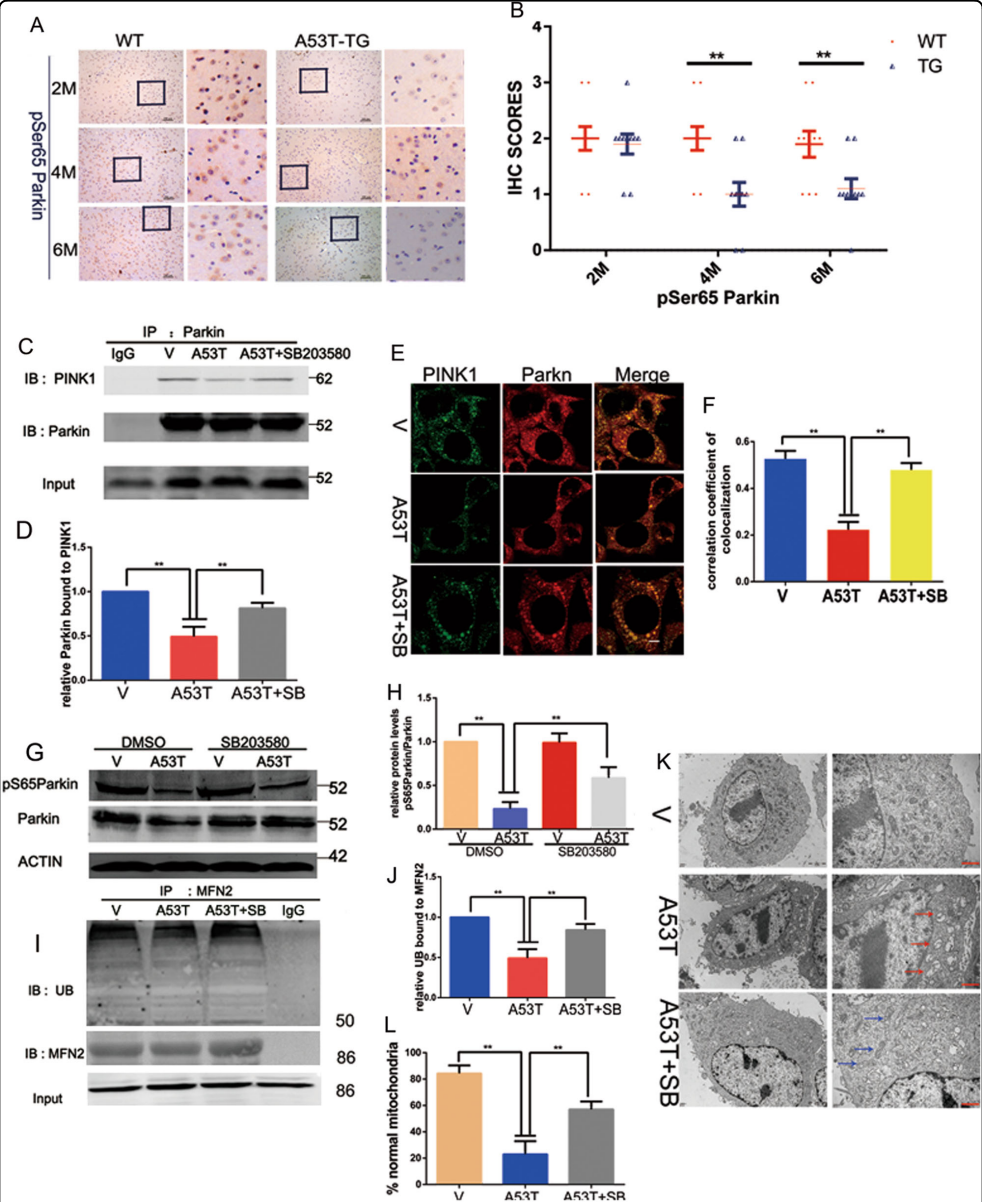
SB203580 treatment inhibits α-synuclein accumulation induced mitochondrial dysfunction through regulating Parkin activation. (**a**, **b**) Immunohistochemistry (IHC) staining of phosphorylated Parkin of serine 65 in the midbrain of 2, 4, and 6 month α-synuclein^A53T^-tg or wild-type mice. **b** Statistical analysis of the average score of phosphorylated Parkin of serine 65 staining between SNCA^A53T^-tg and wild-type mice. *P < 0.05 (Student’s *t*-test). (**c**, **d**) Cell lysates were used for IP with anti-Parkin. Immunoprecipitates or Input were subjected to IB analysis with the indicated antibodies. IgG worked as an immunological control and presented in (**d**). Mean ± SEM. *n* = 3. *p < 0.05. (**e**, **f**) SN4741 cells were subjected to imumunofluorescent co-localization analysis with anti-Parkin (green) and anti-PINK1 (red) and detected under confocal microscope and presented in (**f**). Mean ± SEM. *n* = 10. *p < 0.05. The provided Scale bar in merge image represent 5 μm. (**g**, **h**) Cell lysates were subjected to immunoblotting analysis with anti-Parkin and phospho-Parkin-ser65 and presented in (**h**). Actin was used as loading control. Mean ± SEM. *n* = 3. *p < 0.05. (**i**, **j**) Cell lysates were used for IP with anti-MFN2. Immunoprecipitates or input were subjected to immunoblotting analysis with the indicated antibodies and presented in (**j**). IgG worked as an immunological control. Mean ± SEM. *n* = 3. *p < 0.05. (**k**, **l**) Transmission electron microscopy (TEM) was used to observe the mitochondrial morphology. Swollen mitochondria appeared fractured cristae (red arrow) and α-synucleinA53T SN4741 cells treated with SB203580 appeared intact cristae and normal morphological characteristics(blue arrow). The data was presented in (**l**). Mean ± SEM. *n* = 10. *p < 0.05. The provided Scale bar in merge image represent 2 μm

Parkin works as a vital role in clearing damaged mitochondria by PINK1–Parkin pathway, and its dysfunction participates in neurodegenerative disease^1,11,12^. It is well-known that Parkin-mediated mitophagy is dependent on interaction with PINK1 on the OMM of depolarized mitochondria, and then PINK1 phosphorylates Parkin at serine 65 to activate Parkin. To determine PINK1–Parkin pathway participate in the stimulation of mitophagy, the phosphorylation of Parkin at serine 65 in the midbrain of 2, 4, and 6 month α-synuclein ^A53T^-tg mice were detected by IHC. Results showed that the phosphorylation of Parkin at serine 65 was decreased in SNpc DA neurons of SNCA^A53T^-tg mice, compared with wild-type mice (Fig. 4a, b). Furthermore, we measured the interaction between PINK1 and Parkin as well as Parkin phosphorylation at serine 65 in SN4741 cells. As expected, we observed that SB203580 restored the reduction of PINK1–Parkin interaction and co-localization (Fig. 4c–f), and increased the level of Parkin phosphorylation at serine 65 (Fig. 4g, h). Meanwhile, the ubiquitylation of MFN2, a substrate of Parkin, were detected (Fig. 4i, j). We also observed mitochondria ultrastructure under TEM (Fig. 4k, l). Mitochondria appeared swollen with disappearance of ruptured cristae in α-synucleinA53T overexpression cells, while the mitochondria in α-synucleinA53T overexpression cells treated with SB203580 appeared intact cristae and normal morphological characteristics. Therefore, we concluded that SB203580 alleviates mitochondria dysfunction, improved the level of mitophagy via PINK1–Parkin pathway in α-synuclein A53T model.

### P38 MAPK interacts with Parkin and phosphorylates Parkin at serine 131

**Fig. 5:**
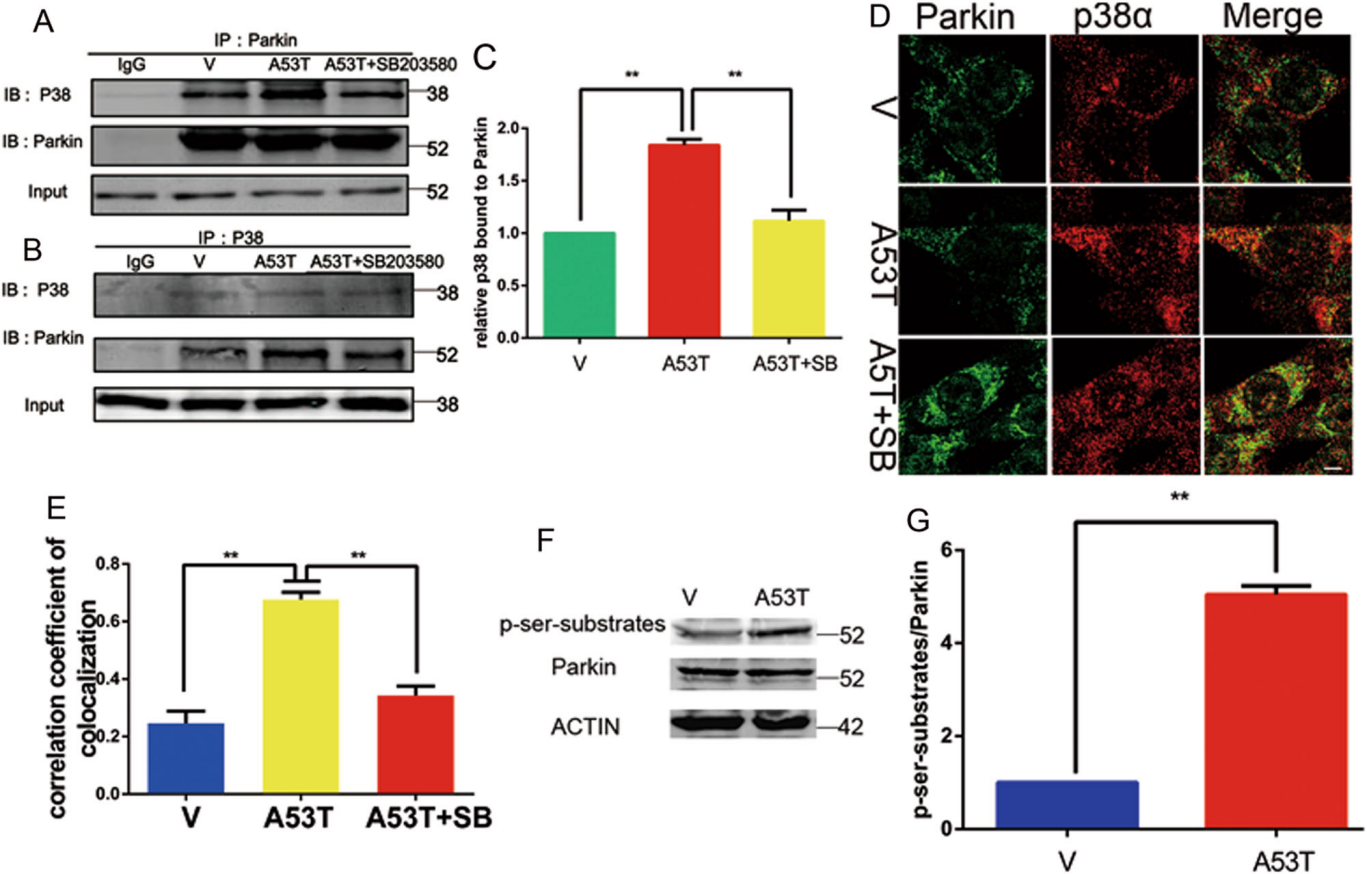
p38 MAPK interacts with Parkin. (**a**–**c**) Cell lysates were used for IP with anti-Parkin or anti-p38, respectively. Immunoprecipitates or Input were subjected to immunoblotting analysis with the indicated antibodies and showed in (**c**). IgG worked as an immunological control. Mean ± SEM. *n* = 3. *p < 0.05. (**d**, **e**) SN4741 cells were subjected to immunofluorescent co-localization analysis with anti-Parkin (green) and anti-p38 (red) and detected under confocal microscope and presented in (**e**). Mean ± SEM. *n* = 10. *p < 0.05. The provided Scale bar in merge image represent 5 μm. (**f**, **g**) Immunoblotting analyzed the level of phosphorylated Parkin from cell lysates by anti-ser-substrates blot and showed in (**g**). Actin was used as loading control. Mean ± SEM. *n* = 3. *p < 0.05

To evaluate whether Parkin could be a substrate of p38 MAPK, we first detected the interaction between p38 MAPK and Parkin by performing co-immunoprecipitation experiments and immunofluorescence microscopy. It revealed that SB203580 decreased the interaction and co-localization that enhanced by α-synucleinA53T aggregation (Fig. 5a–e). We also found that α-synucleinA53T causes an increase in phosphorylated serine signal migrating at Parkin position (Fig. 5f, g).

**Fig. 6:**
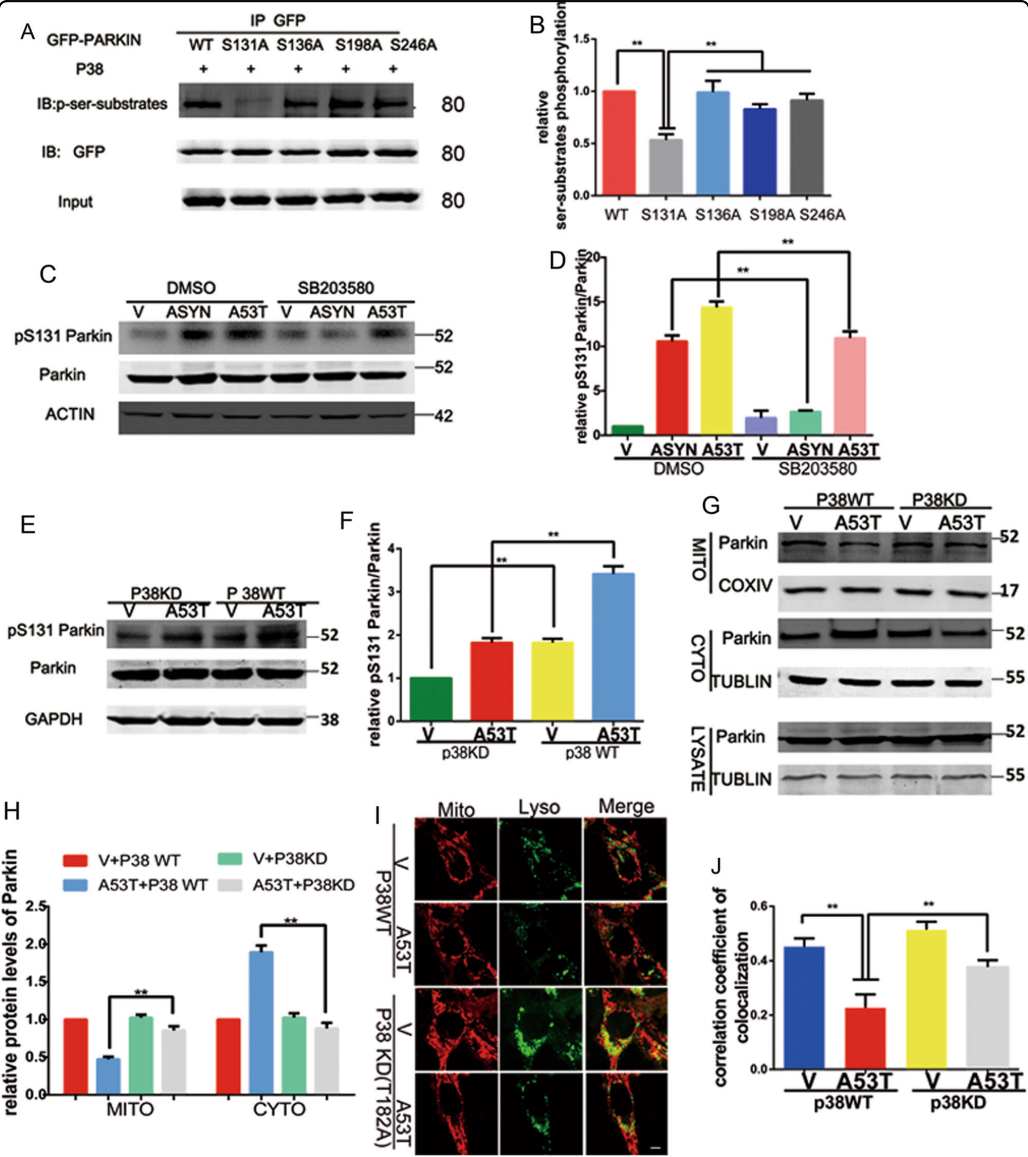
p38 MAPK phosphorylates Parkin at serine 131. (**a**, **b**) SN4741 cells were transfected with wild-type EGFP-Parkin or its point-mutants (EGFP-Parkin S131A, EGFP-Parkin S136A, EGFP-Parkin S198A, EGFP-Parkin S246A) and p38 MAPK WT. Cell lysates were subjected to IP with anti-GFP antibody, followed by immunoblotting with anti-ser-substrates and anti-GFP antibody, Input were subjected to immunoblotting analysis with anti-GFP antibody and presented in (**b**). Mean ± SEM. *n* = 3. *p < 0.05. (**c**, **d**) SN4741 cells transfected with empty vector, **α**-synuclein WT, **α**-synuclein A53T were treated with DMSO or SB203580 (10 μM, 24 h). After treatment, the level of Parkin and phospho-Parkin-ser131 were detected by immunoblotting and presented in (**d**). Actin was used as loading control. Mean ± SEM. *n* = 3. *p < 0.05. (**e**–**j**) SN4741 cells were transfected with empty vector, **α**-synuclein A53T, p38 MAPK WT, or kinase-dead p38 MAPK (T182A) in combination. (**e**) The level of Parkin, phospho-Parkin-ser131, were detected by immunoblotting and presented in **f**. GAPDH was used as loading control. Mean ± SEM. *n* = 3. *p < 0.05. **g** The level of Parkin in the mitochondria component, cytosolic fractions, and total cell lysates were measured by the indicated antibodies and presented in **h**. Tublin was used as loading control in total cell lysates and cytosolic fractions, COXIV was used as loading control in mitochondria. Mean ± SEM. *n* = 3. *p < 0.05. (**i**, **j**) The co-localization analysis of mitochondria and lysosome were examined by confocal microscope and showed in **j**. Mean ± SEM. *n* = 10. *p < 0.05. The provided Scale bar in merge image represent 5 μm

According to analysis of peptide sequence of Parkin and p38 MAPK recognizes substrate proteins, we found that Parkin has four putative p38 MAPK phosphorylation sites in Parkin at serine 131, serine136, serine198, and serine246. To identify which residues of Parkin that are phosphorylated by p38 MAPK, four full-length EGFP-Parkin constructs with point-mutations by alanine substitution were generated (EGFP-Parkin S131A, S136A, S198A, and S246A). After SN4741 cells were co-transfected with Parkin mutants and p38 MAPK, we measured the level of phosphorylation with p-serine-substrates antibody. The results showed that the EGFP-Parkin S131A mutant had a lower phosphorylation level than the other Parkin mutants (Fig. 6a, b). This implied that p38 MAPK phosphorylated Parkin mainly at serine 131. To verify the occurrence of the phosphorylation, we detected the level of Parkin phosphorylation with anti-phosphor-Parkin (ser131) antibody. It showed that SB203580 decreased the Parkin phosphorylation at serine 131 (Fig. 6c, d). Moreover, we transfected SN4741 cells with either kinase-dead p38 MAPK construct (T182A) or p38 MAPK WT construct, as shown in Fig. 6e. Kinase-dead p38 MAPK (T182A) abolished the Parkin phosphorylation at serine 131 in response to α-synuclein accumulation (Fig. 6e, f). To confirm the mechanism of p38 MAPK in regulation of Parkin’s function, we analyzed the effect of overexpression kinase-dead p38 MAPK mutant on Parkin’s translocation to mitochondria. Compared with p38 MAPK WT, kinase-dead p38 MAPK overexpression increased Parkin’s translocation to mitochondria (Fig. 6g, h). Meanwhile, the kinase-dead p38 MAPK enhanced the co-localization of mitochondria and lysosome (Fig. 6i, j). These results supported that p38 MAPK activation suppresses mitophagy via phosphorylating Parkin at serine 131, and then decreases its interaction with PINK1, reduces Parkin’s activation.

### EGFP-Parkin S131A mutant enhances Parkin E3 ligase activity

**Fig. 7:**
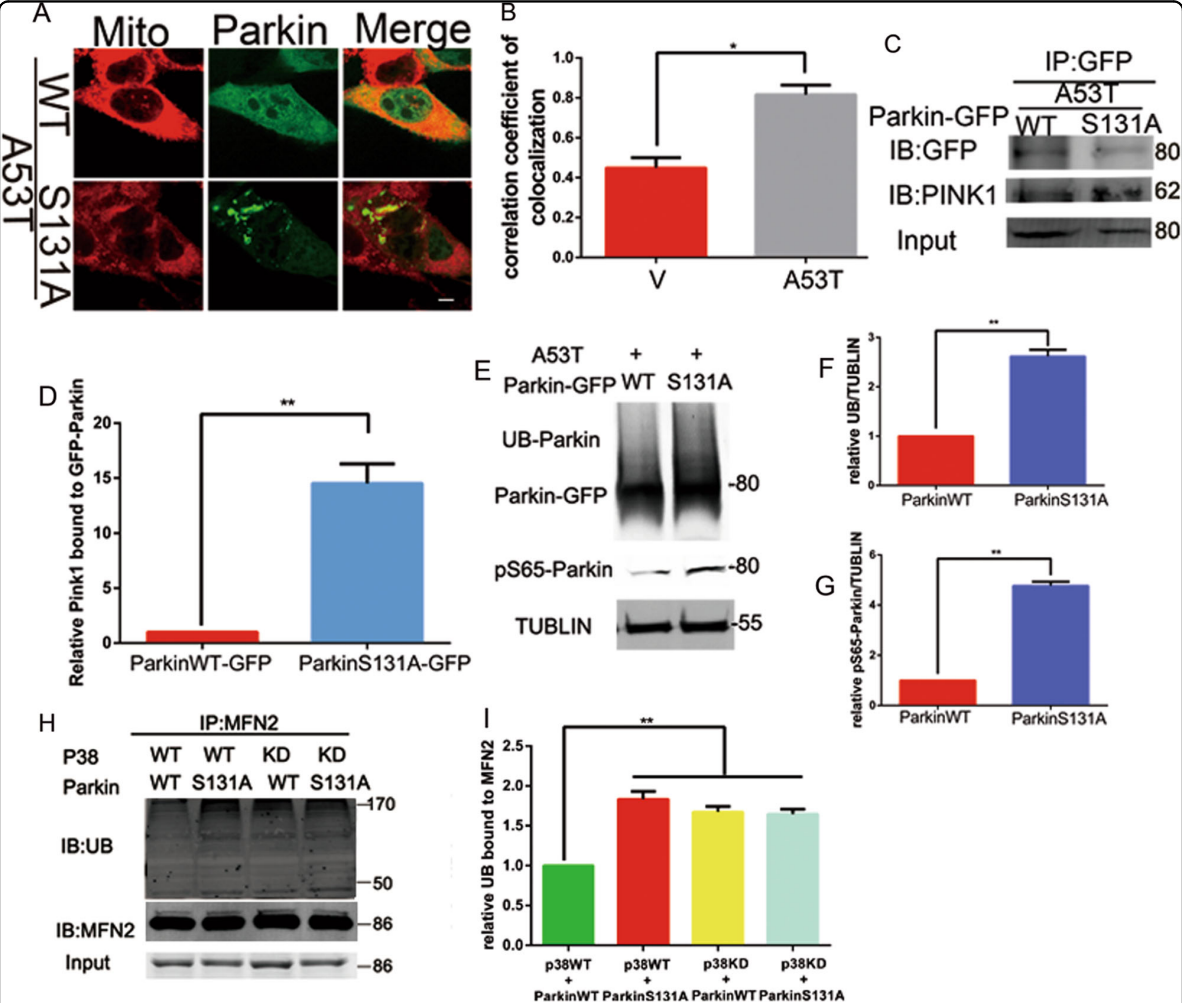
EGFP-Parkin S131A mutant enhances Parkin E3 ligase activity through PINK1–Parkin pathway. (**a**–**h**) SN4741 cells were transfected with EGFP-Parkin S131A and EGFP-Parkin WT together with **α**-synuclein A53T. (**a**) The co-localization analysis mitochondria and EGFP-Parkin WT or EGFP-Parkin S131A and presented in **b**. Mean ± SEM. *n* = 10. *p < 0.05. The provided Scale bar in merge image represent 5 μm. **c** Cell lysates were used for IP with anti-GFP antibody, immunoblotting detected with anti-PINK1 and anti-GFP antibody, Input were detected with anti-GFP antibody and presented in **d**. Mean ± SEM. *n* = 3. *p < 0.05. **e** Cells lysates were subjected to immunoblotting analysis with Parkin, anti-pSer65Parkin. Tublin was used as loading control. The data was presented in (**f**) and (**g**. **h**) Cell lysates were used to for IP with anti-MFN2 antibody, immunoblotting detected with anti-UB and anti-MFN2 antibody, Input were detected with anti-MFN2 antibody. The data was presented in **i**. Mean ± SEM. *n* = 3. *p < 0.05

To further determine the consequences of Parkin serine 131 phosphorylation, we performed immunofluorescence and co-immunoprecipitation. In contrast with EGFP-Parkin WT, EGFP-Parkin S131A increased its translocation to mitochondria (Fig. 7a, b). Besides, EGFP-Parkin S131A significantly increased the interaction between Parkin and PINK1 (Fig. 7c, d). Meanwhile, EGFP-Parkin S131A enhanced Parkin’s autoubiquitination and serine 65 phosphorylation (Fig. 7e–g). We also transfected with p38 MAPK, kinase-dead p38 MAPK (T182A) mutant, EGFP-Parkin WT, EGFP-Parkin S131A in combination, co-immunoprecipitation of MFN2, to determine MFN2-ubiquitination. Immunoblot revealed that both Parkin S131A and kinase-dead p38 MAPK (T182A) increased the level of MFN2-ubiquitination compared to the wild-type group (Fig. 7h, i). These data in total further identified that p38 MAPK-mediated phosphorylation of Parkin at serine 131 decreases its E3 ligase activity via impairing PINK1–Parkin pathway.

### SB203580 reverses synaptic loss and increases TH level

**Fig. 8:**
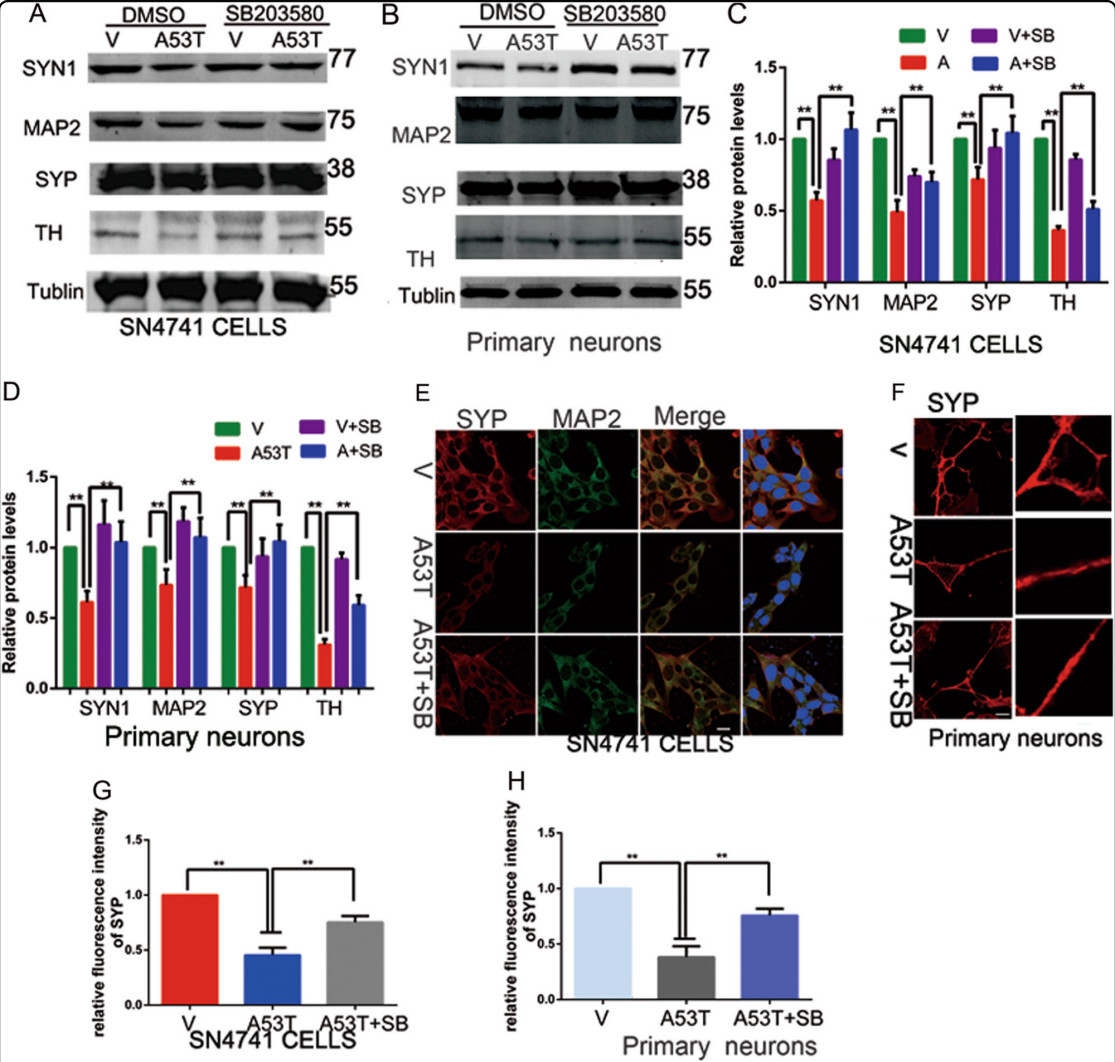
SB203580 reverses synaptic loss and increases TH level. (**a**–**d**) SN4741 cells and primary midbrain neurons derived from postnatal day 0 mouse pups transfected with empty vector, α-synuclein A53T were treated with SB203580 or DMSO. Cells lysates were subjected to immunoblotting analysis with anti-synaptophysin, anti-synapsin-1, anti-MAP2, and TH. The data presented in **c** and **d**, mean ± SEM. *n* = 3. *p < 0.05. **e** SN4741 cells were subjected to immunofluorescent staining for presynaptic marker SYP (red) and dendritic marker MAP2 (green) and presented in **g**. The provided Scale bar in merge image represent 10 μm. Mean ± SEM. *n* = 10. *p < 0.05. **f** Primary midbrain neurons derived from postnatal day 0 mouse pups in cultures were subjected to immunofluorescent staining for presynaptic marker SYP (red) and presented in **h**. The provided Scale bar in merge image represent 5 μm. Mean ± SEM. *n* = 10. *p < 0.05

Synapse loss correlates with cognitive impairment in neurodegenerative disease, including AD and PD^13,14^. To further identify the protective effect of p38 MAPK inhibition in neurodegeneration of PD, we detected the presynaptic proteins synapsin-1(SYN-1) and synaptophysin (SYP), MAP2, and TH. Primary SNpc neurons of mouse were infected with adenovirus that expression either GFP, α-synuclein A53T at 7 DIV. And neurons were used to preincubated with p38 MAPK inhibitor SB203580(24 h, 10 μM) at 8 DIV (days in vitro) and the cultures were analyzed at 10 DIV. As Fig. 8a–d shown that α-synucleinA53T overexpression decreased the level of SYP, SYN1, MAP2, and TH, while SB203580 treatment restored this effect both in SN4741 cells and primary SNpc neurons of mice. The results of immunofluorescence revealed that α-synucleinA53T overexpression decreased the synaptic density based on the fluorescence intensity of SYP, and SB203580 rescued the effect (Fig. 8e–h). These data implied that p38 MAPK inhibitor SB203580 prevents progressive synapse loss in PD.

## Discussion

Mitochondrial dysfunction and α-synuclein abnormal accumulation are widely considered to play critical roles in the underlying mechanisms contributing to degeneration in PD. In this study, we identify p38 MAPK acts as the upstream of Parkin and negatively regulates Parkin’s activity via phosphorylation, revealing that p38 MAPK activation leads to cell death and synapse loss through disrupting mitophagy. These findings help to understand the relationship between the two central pathogenic mechanisms: mitochondrial dysfunction and α-synuclein abnormal accumulation, and provide a potential treatment strategy.

p38 MAPK, a specific class of serine/threonine kinase, is implicated in the process of neurodegenerative disease^3,15,16^. Previous reports suggested that lysosomal deficiency is evident in vitro models and rodent models of PD, as well as in human PD. Prevailing published evidence showed that the PI3K complex and mTOR pathways, modulate macroautophagy^17,18^. Less is known about as to molecular mechanism how mitophagy is regulated by other kinases except for PINK1–Parkin pathway. Hwang et al. have reported that p38 MAPK activation inhibits autophagy and causes mitochondrial impairment by an unknown mechanism in PD^3^. Here, we provide evidence that Parkin acts as a novel substrate of p38 MAPK in the process of α-synucleinA53T-induced mitochondrial dysfunction and neuron degeneration. Our study showed that p38 MAPK activation leads to mitochondrial dysfunction and mitophagy impairment may be a core mechanism underlying α-synuclein accumulation associated PD pathogenesis for following reasons. First, p38 MAPK was activated both in ventral midbrain of α-synuclein^A53T^-tg mice and in α-synuclein^A53T^-tg primary neurons (Fig. 1), which correlates with mitochondrial dysfunction^19^. Second, p38 MAPK inhibition significantly restored mitochondrial membrane potential loss (Fig. 3a), promoted the formation of mitochondrial autolysosomes (Fig. 3g) and increased the number of mitochondria with intact cristae and normal morphological characteristics (Fig. 4k). Third, p38 MAPK activation induces apoptosis through mitochondrial death pathway, which promotes neuronal degeneration and cell death^20,21^. Finally, p38 MAPK negatively regulated Parkin activity, which disrupted mitophagy (Fig. 4). p38 MAPK activation correlated well with Parkin inactivation both in ventral midbrain of α-synuclein^A53T^-tg mice and α-synucleinA53T SN4741cells (Figs. 1 and 4). In this study, we provide evidence for p38 MAPK activation causes mitophagy impairment, which promotes neuronal apoptosis and synaptic loss in α-synucleinA53T SN4741 cells. Various α-synuclein transgenic mice with different promoter are now available. M83 line, the α-synuclein^A53T^-tg mice used in this study, with mouse prion protein promoter has many similarities with human neuronal deficits. Giasson et al. identified that the motor impairment and age-dependent intracytoplasmic neuronal α-synuclein inclusions in the M83 line^22–25^, while the cell loss in the substantia nigra is controversial. In this study, we found that p38 MAPK activation and α-synuclein accumulation in SNpc of M83 line (Fig. 1a, b and S2D, E). Whether the p38 MAPK activation is an early molecular phenomenon in PD pathophysiology or result in the cell loss in the substantia nigra in vivo is need to identify in other α-synuclein transgenic mice models^26^.

The E3 ubiquitin ligase Parkin plays a key role in mitophagy, maintaining mitochondrial health^27,28^. The activity of Parkin is regulated by post-translational modifications especially phosphorylation^29^. PINK1-dependent phosphorylation of Parkin at serine 65 results in its translocation and mediates mitophagy^27^. On the contrast, phosphorylation of Parkin by CK1, C-ABL, and CDK5 lead to the inactivation of Parkin^4,30,31^. A previous study reported that CDK5 phosphorylated Parkin at serine 131 in HEK293 cells^30^, while our results shown CDK5 inhibitor Dinaciclib can not affect the level of phosphorylated Parkin in α-synucleinA53T SN4741 cells (figure S1E). Here we describe a novel mechanism of Parkin phosphorylation in PD. In our experiments, p38 MAPK inhibitor SB203580 and kinase-dead p38 MAPK overexpression decreased serine 131 phosphorylation and increased serine 65 phosphorylation in α-synucleinA53T SN4741, indicating that a crosstalk presented between serine 65 and serine 131 phosphorylation in the regulation of Parkin function. Indeed, Parkin serine 65 phosphorylation decreased in α-synuclein^A53T^-tg mice (Fig. 4a, b). α-synucleinA53T overexpression increased PINK1 (figure S1D), decreased Parkin’s mitochondrial translocation (Fig. 2) and its serine 65 phosphorylation (Fig. 4). Moreover, EGFP-ParkinS131A overexpression markedly increased PINK1-mediated serine 65 phosphorylation and MFN2 ubiquitination (Fig. 7f, i). This data suggest that pSer131 Parkin disrupts PINK1–Parkin pathway. The underlying mechanism PINK1 cannot recognize and recruit pSer131 Parkin need to be further explored. Parkin serine 131 phosphorylation changes Parkin’s molecular structure may be a possible explanation^32^. Together, these results uncovered that p38 MAPK-mediated Parkin serine 131 phosphorylation negatively regulates mitophagy via disrupting PINK1–Parkin pathway.

Mitophagy plays a core role in mitochondrial clearance and quality control through the lysosomal machinery^27,33^. Our results identify that p38 MAPK inhibitor protects neuron degeneration via enhancing mitophagy. In addition, inhibition of p38 MAPK kinase activity rescues synaptic impairment through increasing expression levels of presynaptic proteins, and TH.

Together, our results provided evidences that p38 MAPK negatively regulates the Parkin’s function through phosphorylating its serine 131 residues which induces neuronal cell death and synapse loss in the α-synuclein abnormal accumulation model. The results provide new insight into the role of p38 MAPK activation and mitochondrial clearance machinery in PD. Notably, p38 MAPK inhibition is a potentially valuable strategy to prevent progressive neuronal degeneration via enhancing mitophagy.

## Material and methods

### Plasmids, antibody, and chemicals

EGFP-Parkin WT, EGFP-ParkinS131A, S136A, S198A, S246A were supplied commercially by Obio Technology. Corp. Ltd. (Shanghai, China). Other constructs were: EGFP-α-synuclein A53T (Addgene plasmid 40823; Addgene), PHM6-α-synuclein-A53T (Addgene plasmid 40825; Addgene), pAAVα-synuclein WT (Addgene plasmid 36055; Addgene), EGFP-Parkin WT (Addgene plasmid 45875; Addgene), PCDNA3 Flag p38 alpha (Addgene plasmid 20352; Addgene), pMT3-p38 (Addgene plasmid 12658; Addgene). The following antibodies were used: anti-Parkin #4211(Cell Signaling Technologies, Danvers, MA, USA,), anti-LC3A/B #4180(Cell Signaling Technologies, Danvers, MA, USA), anti-beta Tubulin #2146 (Cell Signaling Technologies, Danvers, MA, USA), anti-p38 ab170099 (Abcam, Cambridge, UK), anti-p-p38 tyr182 sc-166182 (Santa Cruz Biotechnology, Santa Cruz, CA, USA), anti-PINK1 #6946 (Santa Cruz Biotechnology, Santa Cruz, CA, USA), anti-COXIV sc-69360 (Santa Cruz Biotechnology, Santa Cruz, CA, USA), anti-MFN2 12186-1-AP (Proteintech, Rosemont, IL, USA), anti-α-synuclein 10842-1-AP (Proteintech, Rosemont, IL, USA), anti-VDAC1 sc-8829 (Santa Cruz Biotechnology, Santa Cruz, CA, USA), anti-p-Parkin ser131 ab12371 (Absci, MD, USA), anti-p-Parkinser65 bs-19882R (Bioss, Beijing, China), anti-UB sc-9133 (Santa Cruz Biotechnology, Santa Cruz, CA, USA), anti-synaptophysin 60191-1-Ig (Proteintech, Rosemont, IL, USA), anti-synapsin-1 #5297 (Cell Signaling Technologies, Danvers, MA, USA), anti-MAP2 #8707 (Cell Signaling Technologies, Danvers, MA, USA). Phospho-MAPK/CDK Substrates (PXS*P or S*PXR/K) #2325 (Cell Signaling Technologies, Danvers, MA, USA). MAPK Family Antibody Sampler Kit #9926 (Cell Signaling Technologies, Danvers, MA, USA). SB203580 (S1076), U0126 (S1102), SP600125 (S1460), Dinaciclib (S2768) were obtained from Selleckchem (Houston, TX, USA).

### Animal

The α-synuclein^A53T^-tg mice expressed mutant human A53T α-synuclein were purchased from Model Animal Research Center of Nanjing University. The α-synuclein^A53T^-tg mice express mutant A53T α-synuclein (140 amino acid isoform) under the direction of the mouse prion protein promoter has been discribed^22^. Mice expressing A53T α-synuclein (line M83) is one of the early-onset PD models. The transgenic mice are generally used in studies of therapeutic targets and mechanisms of Parkinson’s Disease. Animals were individually housed under light:dark (12:12 h) cycles and provided with food and water.

### Cell culture and transfection

SN4741 cells were cultured in DMEM medium containing 1% glucose, 100 U/ml penicillin-streptomycin, and l-glutamine and supplemented with 10% fetal bovine serum at 33 °C. Cells were transfected with Lipofectamine 3000 reagent (Invitrogen, Carlsbad, CA, USA) according to manufacturer’s instructions.

### Primary midbrain dopaminergic neuron

Primary midbrain dopaminergic neurons were derived from ventral mesencephalon of postnatal day 0 mouse pups based on Florence Gaven’s protocol^34^. In brief, dissociated cells were seeded at cell culture dishes coated with poly-d-lysinecoated. Cells were maintained at 37 °C in a humidified atmosphere of 5% CO2 and 95% air in DMEM/F12 medium containing 10% fetal bovine serum, 50 U/ml penicillin, and 50 g/ml streptomycin. Next day, culture medium was changed to B-27 Plus Neuronal Culture System (Thermo Fisher Scientific, A3653401) containing 50 U/ml penicillin, and 50 g/ml streptomycin. At DIV 7, DA neurons were detected by anti-TH antibody with immunofluorescence (fig. S2B). Seven-day-old cultures were used for treatment. Adenovirus that expression either GFP, α-synuclein A53T were used to infect Primary midbrain dopaminergic neuron at 7 DIV.

### Western blot and co-immunoprecipitation

Cytosolic and mitochondrial fractions were collected using a mitochondrial isolation kit (KeyGEN, Nanjing, China, KGA827) following the manufacturer’s instructions. Cells were collected in a lysis buffer containing 20 mM Tris-HCl, pH 7.4, 150 mM NaCl, and 1% Triton X-100 with protease inhibitor cocktail (Roche, Nutley, NJ, USA). Equal protein concentrations were loaded onto 10% or onto 15% polyacrylamide gels. Gels were transferred onto Hybond-P PVDF membranes. Blots were blocked in 5% nonfat dry milk for 1 h at room temperature and then incubated overnight with the primary antibodies. The membranes were incubated with secondary antibody for 1 h at room temperature and the signal was detected by Odyssey Infrared Imaging System. For co-immunoprecipitation, centrifuge at 13,000 × *g* for 10 min at 4 °C, and then transfer the supernatant to a new centrifuge tube. Add 5 μg of the corresponding antibody to the sample overnight at 4 °C. Next day, add 100 µl agarose beads to the sample 2 h at 4 °C. The immunocomplex were collected with centrifugation at 1500 × *g* at 4 °C for 1 min. Discard the supernatant, the beads were washed 3 times with RIPA. Proteins were eluted from beads with 2× SDS loading buffer. At last, the samples were heated and subjected to immunoblot analysis. The immuno-reactive bands were detected by Odyssey Infrared Imaging System. The band intensity was analyzed with image J imaging software.

### Immunofluorescence

For Immunofluorescence studies, cells were grown on glass coverslips for 2 days, and then cells were washed with 1× PBS three times, then fixed with 4% paraformaldehyde for 15 min at room temperature. Cells were then passed through frozen methanol for 10 min at 20 °C and blocked in 5% BSA for 30 min. slides Incubated with primary antibody (1:100–1:200) in 5% BSA, overnight at 4 °C, and then incubated secondary antibody (anti-mouse and anti-rabbit Alexa Fluor 488 and 594 antibodies (Thermo Fisher) (1:100) in 5% BSA for 60 min at room temperature. An Olympus FV1000 confocal microscope with a ×60 objective was used for taking images. The co-localization was analyzed with image pro plus software.

### Immunohistochemistry

The paraffin sections of bran tissue were collected for routine immunohistochemistry (IHC) staining for p-p38 sc-166182 (Santa Cruz Biotechnology, Santa Cruz, CA, USA) and pS65Parkin MBS8509495 (Mybiosource, CA, USA). The area of the midbrain dopaminergic neurons of brain sections according to the anatomical position and the location of TH-positive neurons (fig. S2A, B). The expression levels of proteins in the brain tissue were scored according to intensity and the percentage of positive cells and their staining intensity as previously described^35^. Specifically, percentages ≤1, 2–25, 26–50, 51–75, and ≥75% were scored as 0, 1, 2, 3, and 4, respectively; non-significant brown, slight brown, moderate brown, and deep brown staining intensities were scored as 0, 1, 2, and 3, respectively. Combined scoring was then calculated and graded as negative (−; 0–1), weak (+; 2–4), moderate (++; 5–8), and strong (+++; 9–12).

### Fluorescent staining of mitochondrial and lysosome

Mitochondrial and lysosome staining was carried as the manufacturer’s instructions. In brief, SN4741 cells were plated into confocal Petri dish. They were fluorescently labeled with 10 nM Mito Tracker Red CMXRos (Life Technologies, M7512) in cell culture medium for 25 min and 0.01% Lyso Tracker Green DND-26 (Life Technologies, L7526) in cell culture medium for 25 min at 33 °C. After washing with medium, living cells were analyzed for mitochondrial morphology and co-localization between mitochondrial and lysosome by laser scanning confocal microscopy.

### Measurement of mitochondrial membrane potential

The mitochondrial membrane potential (ΔΨm) in cells was assessed using 5,5,6,6-tetrachloro-1,1,3,3-tetraethylbenzimidazolyl-carbocyanine iodide((JC-1; Molecular Probes, Inc., Eugene, OR, USA). Cells were washed with PBS and incubated at 33 °C for 15 min with 6.5 μM JC-1 in serum-free media in the dark. J-aggregates were monitored using laser scanning confocal microscopy.

### Transmission electron microscopy (TEM)

SN4742 cells were treated and then washed once in PBS, collected with cell scraper, resuspended in a fixative containing ice-cold 2.5% glutaraldehyde in PBS at 4 °C, and centrifuged. Cell pellets were fixed for 3 h. After rinsing with sodium cacodylate buffer, the cell pellets was further fixed in 1% OsO4 in sodium cacodylate buffer on ice for 1 h and dehydrated with acetone. The cell pellets was embedded in resin and polymerized at 60 °C for 48 h. Ultrathin sections were obtained on ultramicrotome and stained with uranyl acetate and lead citrate before observation under Hitachi H-7500 TEM.

### Statistical analysis

All experiments were analyzed using GraphPad Prism 6 software(La Joya, Ca, USA). Data were analyzed with Student’s *t*-tests or ANOVA with Tukey’s post-test of multiple comparisons, and statistical significance is expressed as *P < 0.05. **P < 0.01. All experiments were repeated at least three times and all values were described as mean ± SEM.

## Electronic supplementary material

Supplementary Figure legends

Figure S1

Figure S2
