# Supplementary material for: Phosphorylation of Parkin at serine 131 by p38 MAPK promotes mitochondrial dysfunction and neuronal death in mutant A53T α-synuclein model of Parkinson’s disease
Source: Cell Death Dis. 2018 Jun 13;9(6):700. doi: 10.1038/s41419-018-0722-7 (PMC5999948; doi:10.1038/s41419-018-0722-7)
Supplement: Supplementary file 1 — Supplementary Figure legends [file 41419_2018_722_MOESM1_ESM.docx]

**Figure S1** (A)Tail DNA isolated from SNCA^A53T^-tg or wild type mice. The mRNA of α-synucleinA53T was detected by PCR. (B, C) Effiency of α-synucleinA53T overexpression was measured by western blot and show in C. (D) Phosphorylated Erk and JNK, PINK1, caspase 3 were measured by western blot. (E, F) SN4741 cells were treated with Erk inhibitor U0126 or CDK5 inhibitor dinaciclib, and Parkin phosphorylation at serine 65 was measured by western blot and shown in F. (G, H) SN4741 cells were treated with p38MAPK inhibitor SB203580 and the mRNA of α-synucleinA53T were measured by PCR and shown in H. (I) Graphical Abstract.

**Figure S2** (A) Model of anatomical position of midbrain. (B) Immunohistochemistry (IHC) staining of TH in the midbrain of mice. The provided Scale bar in image represent 100μm. (C) Primary midbrain dopaminergic neurons from mice in cultures. Cultures were grown in vitro, fixed, and immunostained for TH. The provided Scale bar in image represent 10μm. (D) Immunohistochemistry (IHC) staining of α-synuclein in the midbrain of 2, 4 and 6 month SNCA^A53T^-tg or wild type mice. The provided Scale bar in image represent 100μm. (E) Statistical analysis of the average score of α-synuclein staining between SNCA^A53T^-tg and wild type mice. **P*<0.05 (Student’s t-test).
