# Supplementary figures and images for: Phosphorylation of Parkin at serine 131 by p38 MAPK promotes mitochondrial dysfunction and neuronal death in mutant A53T α-synuclein model of Parkinson’s disease
Source: Cell Death Dis. 2018 Jun 13;9(6):700. doi: 10.1038/s41419-018-0722-7 (PMC5999948; doi:10.1038/s41419-018-0722-7)

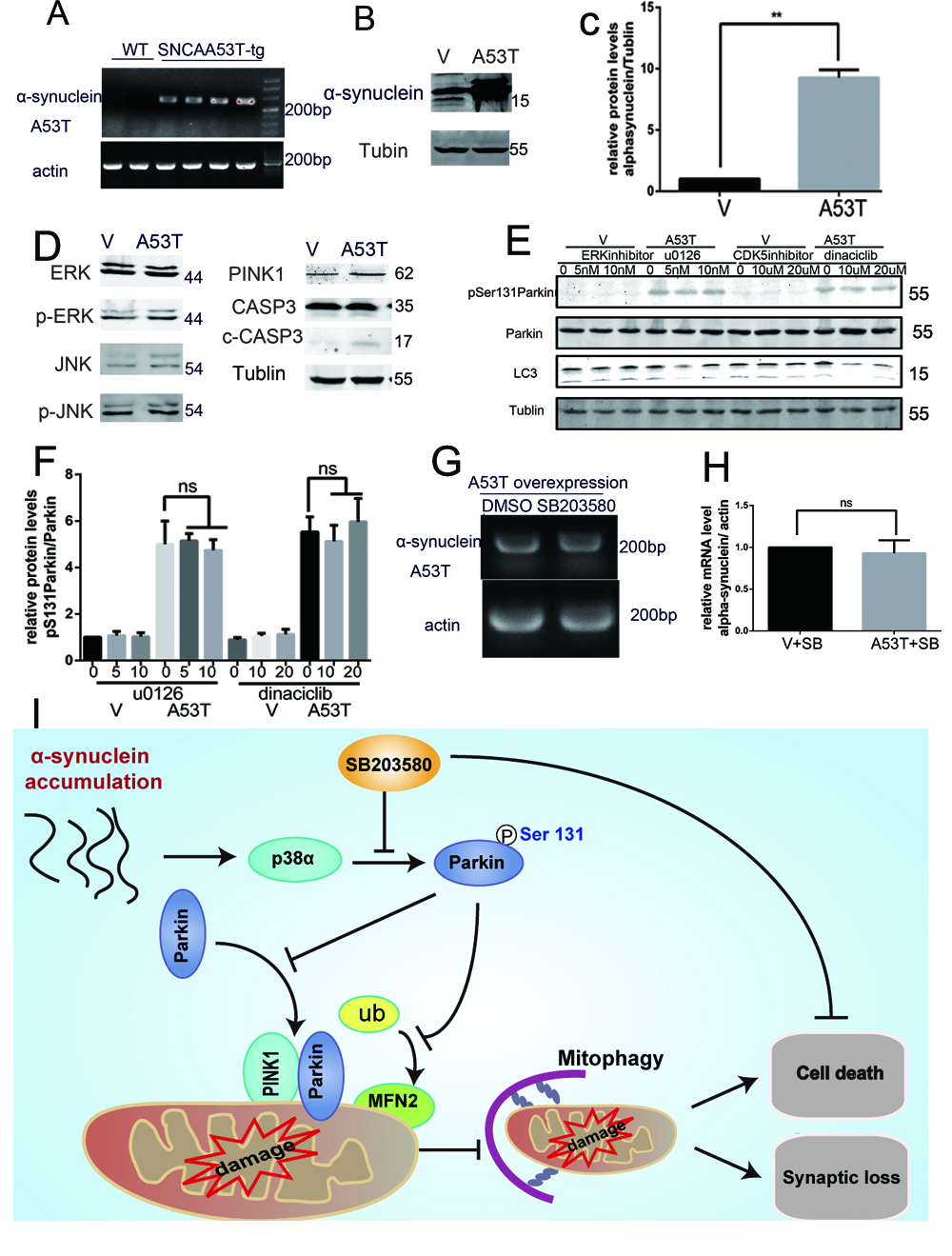

Supplement: Supplementary file 2 — Figure S1 [file 41419_2018_722_MOESM2_ESM.tif]

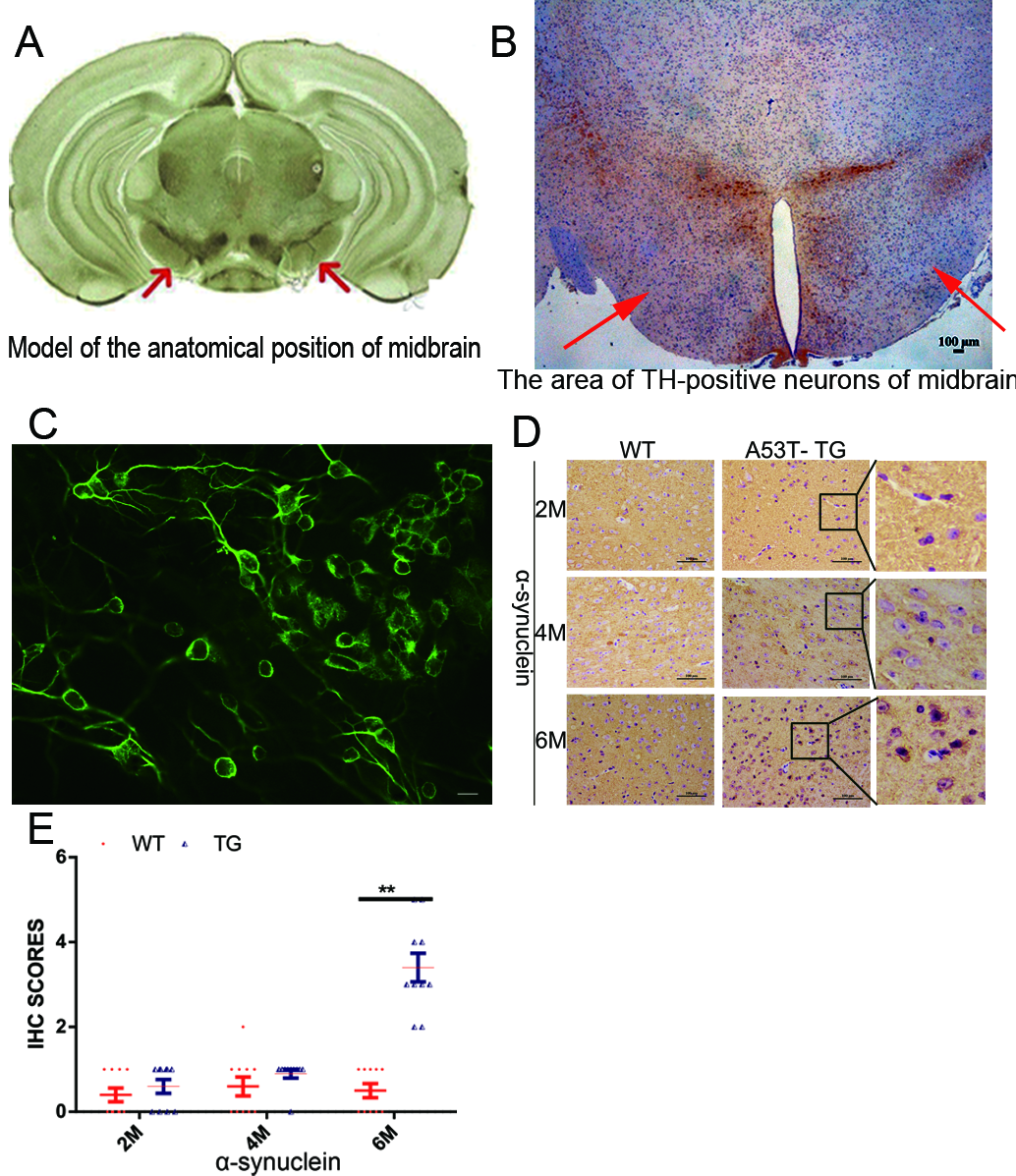

Supplement: Supplementary file 3 — Figure S2 [file 41419_2018_722_MOESM3_ESM.tif]
